# Comparisons of the Pharmacokinetic Profile of Four Bioactive Components after Oral Administration of Gan-Sui-Ban-Xia Decoction Plus-Minus Gansui and Gancao Drug Combination in Normal Rats

**DOI:** 10.3390/molecules20059295

**Published:** 2015-05-20

**Authors:** Yang Zhang, Dawei Qian, Ying Pan, Zhenghua Zhu, Jing Huang, Junzuan Xi, Jianming Guo, Xueping Zhou, Gansheng Zhong, Jinao Duan

**Affiliations:** 1 Jiangsu Collaborative Innovation Center of Chinese Medicinal Resources Industrialization, and National and Local Collaborative Engineering Center of Chinese Medicinal Resources Industrialization and Formulae Innovative Medicine, Nanjing University of Chinese Medicine, Nanjing 210023, China; E-Mails: zy91923@163.com (Y.Z.); ptianxie@126.com (Y.P.); 04040416@163.com (Z.Z.); hjsky2010@hotmail.com (J.H.); jzd666@163.com (J.X.); njuguo@njutcm.edu.cn (J.G.); 2 The No.1 Clinical Medical College, Nanjing University of Chinese Medicine, Nanjing 210023, China; E-Mail: zxp@njucm.edu.cn; 3 Basic Medical College, Beijing University of Chinese Medicine, Beijing 100092, China; E-Mail: Zhonggansheng@sohu.com

**Keywords:** Gan-Sui-Ban-Xia Decoction, Gansui and Gancao anti-drug combination, UPLC–TQ/MS, pharmacokinetics influence

## Abstract

Gan-Sui-Ban-Xia Decoction (GSBXD) was first presented by Zhang Zhongjing in the book *Synopsis of Golden Chamber* during the Han Dynasty period. The formula was then used for the treatment of persistent fluid retention with floating pulse in Traditional Chinese Medicine (TCM), which in modern medicine is known as malignant ascites. Here, a rapid liquid chromatography-tandem mass spectrometry (LC-MS/MS) method has been developed for the determination of glycyrrhizinic acid, liquiritin, paeoniflorin, albiflorin after oral administration of GSBXD plus-minus Gansui and Gancao anti-drug combination to investigate the possible pharmacokinetic profile differences of different prescriptions with GSBXD in normal rats. The differences of pharmacokinetic parameters among groups were tested by the Student’s *t*-test with *p* < 0.05 as the level of significance. Significant differences were found between the Gansui and Gancao anti-drug combination and other herbs in GSBXD on pharmacokinetic profile of glycyrrhizinic acid, liquiritin, paeoniflorin and albiflorin. The obtained knowledge might contribute to the rationality of the clinic use of GSBXD and also reveal the compatibility conditions of the Gansui and Gancao anti-drug combination.

## 1. Introduction

Gan-Sui-Ban-Xia Decoction (GSBXD) was first presented by Zhang Zhongjing in the book *Synopsis of Golden Chamber* during the Han Dynasty period. The formula was then used for the treatment of persistent fluid retention with floating pulse in Traditional Chinese Medicine (TCM), which in modern medicine is known as malignant ascites. Malignant ascites is one of the most serious complications of advanced cancer and is a manifestation of end stage events in different kinds of cancers [1,2]. Because of its definite curative effects and less harm to the health, some crude herbs and TCM recipes have a wide clinical application for malignant ascites in China for many years. GSBXD is one of the famous formulae which have a precise effect. The decoction is composed of four crude herbs, including *Euphorbia kansui T.N.Liou exT.P.Wang.* (Gansui), *Glycyrrhiza uralensis Fisch.* (Gancao), *Paeonia lactiflora Pall.* (Baishao), *Pinellia ternata (Thunb.) Breit.* (Banxia), but the clinical application of GSBXD is limited because of the Gansui and Gancao drug combination which is considered to be a unfavorable combination according to the “Eighteen antagonisms” (also known as Shibafan in Chinese) which are the controversial prohibited combinations in traditional Chinese medicine, based on a history of thousands of years of experience so far. Whether and how they could be used in clinic, has been a problem that people have argued about but without reaching a decision.

**Figure 1:**
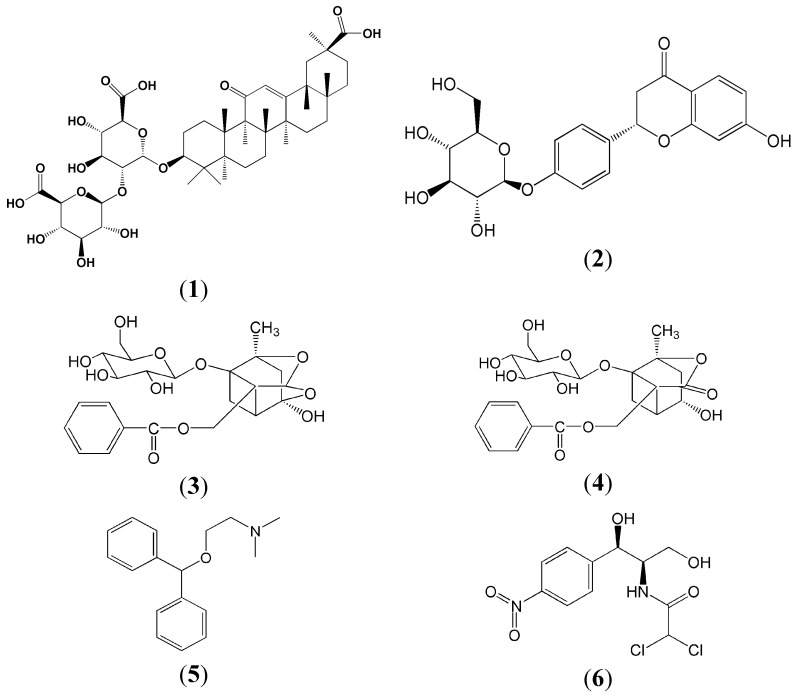
The chemical structures of glycyrrhizinic acid (**1**), liquiritin (**2**), paeoniflorin (**3**), albiflorin (**4**), diphenhydramine (IS for positive ionization mode) (**5**) and chloromycetin (IS for negative ionization mode) (**6**).

Compound traditional Chinese medicines (also known as traditional Chinese formulae) have been used for thousands of years in China and abundant clinical experience about their use has accumulated in long-term clinical practice. TCM has accumulated more than 100,000 formulae over the past 2000 years [3]. A lot of studies have been conducted in the recent past to explore the specificity and regularity of TCMs, among which pharmacokinetic studies on TCMs are useful to evaluate efficacy and predict the safety of TCMs, and can be very helpful to discover the scientific basis for the acxtivity of bioactive components or toxic substances in TCMs [4,5]. Pharmacokinetic studies of many formulae have reported concerns with the drug combinations used in traditional Chinese recipes after oral administration [6,7]. Now in this essay, our study was aimed at creating an effective measure to enhance the safety and reasonable level of the Gansui-Gancao drug combination through the comparison of pharmacokinetic studies of different prescriptions of GSBXD of four compounds, including glycyrrhizinic acid (**1**), liquiritin (**2**), paeoniflorin (**3**), albiflorin (**4**) (Figure 1), These four compounds have been proved to have a number of pharmacological activities, including anti-inflammatory, antiviral, antioxidant, anticancer, antidepressant and liver protection effects [8,9,10,11,12,13,14,15].

In this study, a rapid liquid chromatography-tandem mass spectrometry (LC-MS/MS) method has been developed for the determination of glycyrrhizinic acid, liquiritin, paeoniflorin, albiflorin after oral administration of GSBXD plus-minus Gansui and Gancao anti-drug combination. Meanwhile, we conducted the research to investigate the possible pharmacokinetic profile differences of different prescriptions of GSBXD in normal rats in order to reveal the compatibility conditions of the Gansui and Gancao anti-drug combination and other herbs in GSBXD.

## 2. Results and Discussion

### 2.1. Sample Preparations

Plasma samples (200 μL) and IS solution (50 μL, 1215 ng/mL for diphenhydramine, 476 ng/mL for chloromycetin) were added in an Eppendorf tube, and this mixture was extracted with methanol (550 μL) by shaking on a vortex-mixer for 2 min, and centrifuged for 10 min at 13,000 rpm. The contents were evaporated to dryness on a rotary evaporator at 37 °C. The residue was reconstituted in methanol (100 μL) and centrifuged (13,000 rpm for 10 min). The supernatant was transferred to an auto sample vail and an aliquot of 5 μL was injected onto the UPLC-MS/MS system for analysis.

### 2.2. Method Validation

#### 2.2.1. Specificity

**Figure 2:**
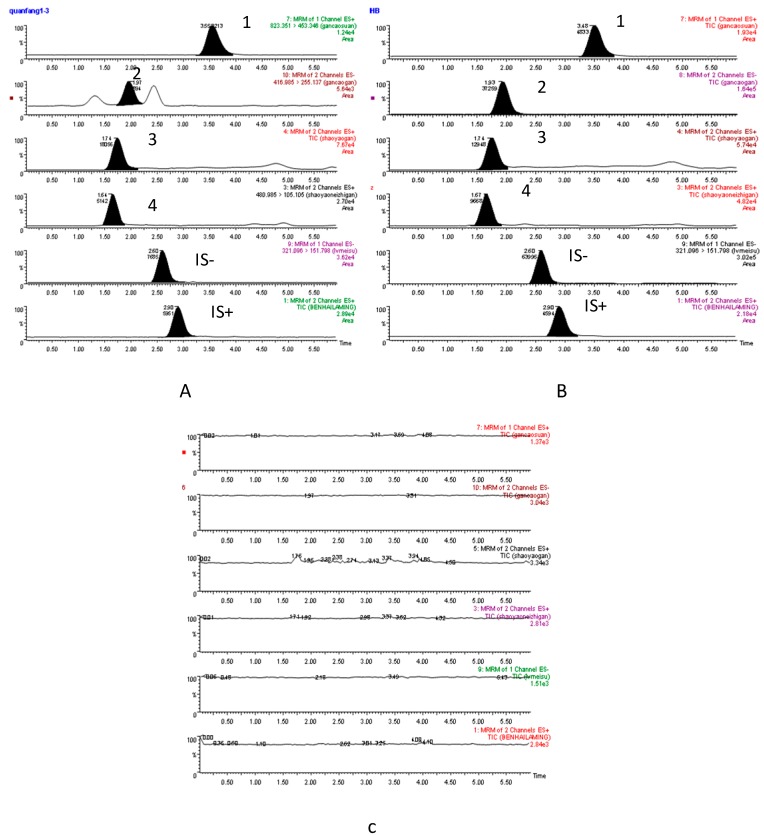
Represent actives extraction MRM chromatograms of compounds **1–4**, chloromycetin (IS-) and diphenhydramine (IS+): (**A**) 15 min sample plasma after a single oral administration of GSBXD; (**B**) blank plasma spiked with the four analytes and IS; (**C**) blank plasma.

All the analytes and internal standard could be detected on MRM spectrograms without any endogenous interference (Figure 2).

#### 2.2.2. Linearity and Lower Limits of Quantification (LLOQ)

The calibration curves of four compounds exhibited good linearity with correlation coefficients (R^2^) within the range from 0.9929 to 0.9993. The LLOQs were suitable for quantitative detection of analytes in the pharmacokinetic studies. Linear ranges, LLOQs, LLODs and correlation coefficients are shown in Table 1.

**Table 1:** The regression equations, linear ranges, LLOQs, and LODs of the four compounds.

Compound No.	Linear Regression Equation	R^2^	Range (ng/mL)	LOD (ng/mL)	LOQ (ng/mL)
**1**	y = 1861.00x + 46.77	0.9929	9.57–2450	2.71	4.50
**2**	y = 14370.00x − 36.39	0.9956	4.32–2210	0.93	1.44
**3**	y = 1076.80x + 32.05	0.9993	8.91–2280	1.66	3.15
**4**	y = 232.76x + 33.35	0.9971	5.53–2830	1.98	2.76

#### 2.2.3. Precision and Accuracy

The results of the intra- and inter-day precision and accuracy of all the analytes in LLOQ and QC samples are summarized in Table 2. The intra- and inter-day precisions ranged 8.19%–12.10% and 8.18%–18.70%, respectively. The accuracy derived from QC samples was between 81.92%–102.21% for each QC level of the four analytes. The results demonstrated that the precision and accuracy values were within the acceptable range.

**Table 2:** Precision and accuracy for the determination of the four compounds in rat plasma.

Compound No.	Concentration (ng/mL)	Intra-day		Inter-day	
		Accuracy (%)	Precision (RSD, %)	Accuracy (%)	Precision (RSD, %)
1	1.91 × 10	90.88	10.60	81.91	8.18
	3.09 × 10^2^	96.45	8.19	99.13	8.91
	1.23 × 10^3^	101.04	8.27	87.58	9.21
2	4.32	90.45	10.56	73.03	18.70
	1.38 × 10^2^	91.22	8.37	99.18	10.22
	1.11 × 10^3^	99.68	10.40	100.09	9.13
3	1.78 × 10	91.09	12.10	80.15	16.41
	1.43 × 10^2^	99.48	9.04	99.28	8.88
	1.14 × 10^3^	102.00	9.13	97.28	10.37
4	1.11 × 10	92.21	8.91	77.59	17.27
	1.77 × 10^2^	99.08	9.33	93.12	10.32
	1.42 × 10^3^	98.90	9.87	99.90	9.49

#### 2.2.4. Extraction Recovery and Matrix Effect

The mean recoveries of all analytes at different concentrations are shown in Table 3. The extraction recoveries of three level QC samples were more than 51.17%.The extraction recovery of IS was 73.04%–102.77%.The matrix effect of blank plasma of all the analytes was found to be within the acceptable range; all values were more than 80.09% (Table 3). The matrix effect of IS was 91.59%–100.01%. Thus, it was demonstrated that the plasma matrix effect was negligible for the assay.

**Table 3:** Recoveries and matrix effects of the four compounds in rat plasma.

Compound No.	Concentration (ng/mL)	Recovery		Matrix effect	
		Accuracy (%)	Precision (RSD, %)	Accuracy (%)	Precision (RSD, %)
**1**	1.91 × 10	71.23	7.19	72.17	12.34
	3.09 × 10^2^	64.32	7.69	89.40	7.53
	1.23 × 10^3^	79.01	5.64	87.08	3.99
**2**	4.32	77.89	13.12	67.16	9.15
	1.38 × 10^2^	81.32	8.90	101.67	8.07
	1.11 × 10^3^	69.68	4.71	80.68	4.93
**3**	1.78 × 10	51.17	17.25	80.09	13.99
	1.43 × 10^2^	99.99	6.44	99.18	6.15
	1.14 × 10^3^	72.93	5.94	96.51	3.01
**4**	1.11 × 10	52.76	11.77	85.63	16.47
	1.77 × 10^2^	69.91	5.24	92.09	4.39
	1.42 × 10^3^	68.01	9.11	94.55	2.13

#### 2.2.5. Stability

Stability of the four analytes during the sample storing and processing procedures was fully evaluated by analysis of QC samples. The results are shown in Table 4. The results indicated that these analytes in rat plasma were all stable for one-month storage at −80 °C, 24 h in the auto-sampler (4 °C) and three freeze-thaw cycles with accuracy in range of 79.13%–104.13%.

**Table 4:** Stability of the four compounds in rat plasma.

Compound No.	Concentration (ng/mL)	Freeze-thaw cycles		At −80 °C for a Month		Autosampler for 24 h	
		Accuracy (%)	Precision (RSD, %)	Accuracy (%)	Precision (RSD, %)	Accuracy (%)	Precision (RSD, %)
**1**	1.91 × 10	98.83	8.10	89.99	4.61	93.94	8.53
	3.09 × 10^2^	91.02	7.10	91.37	8.16	92.23	9.91
	1.23 × 10^3^	94.33	10.22	98.18	9.93	87.36	7.48
**2**	4.32	101.l6	9.83	91.03	6.51	88.92	6.22
	1.38 × 10^2^	89.99	11.03	99.99	7.83	97.49	7.42
	1.11 × 10^3^	95.35	7.59	96.71	10.33	97.11	9.03
**3**	1.78 × 10	87.63	4.81	87.54	9.40	98.03	9.48
	1.43 × 10^2^	92.10	10.47	95.32	6.43	91.28	4.50
	1.14 × 10^3^	91.57	7.89	89.55	8.16	104.13	6.13
**4**	1.11 × 10	93.49	11.36	101.5	10.01	97.15	9.33
	1.77 × 10^2^	102.34	9.68	79.13	3.93	91.22	7.06
	1.42 × 10^3^	98.33	6.77	88.48	6.71	99.91	9.10

### 2.3. Pharmacokinetics Study

**Figure 3:**
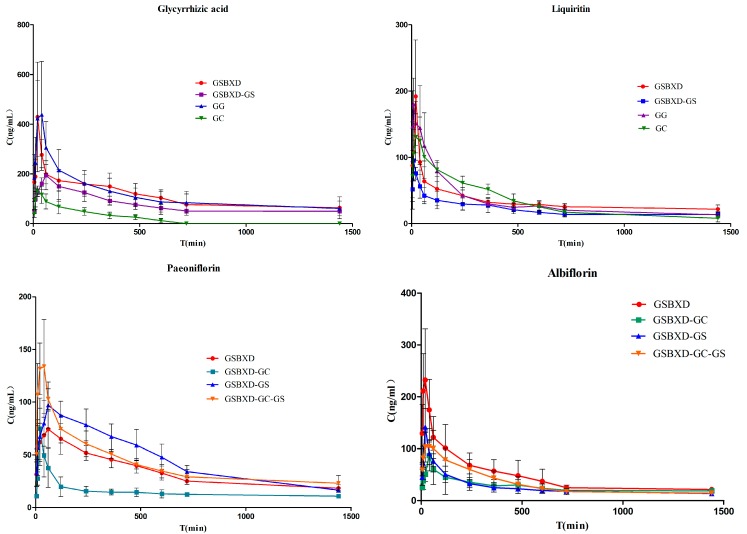
Mean plasma concentration-time curves of four compounds in normal rats (*n* = 6).

The developed and validated method was applied to the pharmacokinetic evaluation of the four compounds after oral administration of different fractions to normal rats (Figure 3). The assay was proved to be sensitive enough for the determination of these analytes in rat plasma. The pharmacokinetic parameters including half-time (*T*_1/2_), maximum plasma concentration (*C*_max_), time to reach the maximum concentrations (*T*_max_), are a under concentration–time curve (*AUC*_0~t_) calculated by non-compartment model are displayed in Table 5. 

**Table 5:** Pharmacokinetic parameters of four compounds after an oral administration in normal rats (*n* = 6).

Compound No.	Group	*C*_max_/ng·mL^−1^	*T*_max_/h	*T*_1/2_/h	*AUC*_0~t_/ng·h·mL^−1^
**1**	GSBXD	503.15 ± 199.16	0.33 ± 0	5.93 ± 2.67	2631.14 ± 531.93
	GSBXD-GS	213.90 ± 55.44 *	1.67 ± 0.12 *	11.56 ± 5.74	1278.93 ± 392.92 **
	GG	364.49 ± 46.19	0.64 ± 0.34	9.29 ± 4.70 **	787.72 ± 109.53 **
	GC	146.86 ± 19.86	0.36 ± 0.16	0.79 ± 0.39	485.51 ± 136.03
**2**	GSBXD	192.59 ± 84.49	0.39 ± 0.14	7.04 ± 2.99	793.58 ± 141.51
	GSBXD-GS	98.93 ± 26.76	0.17 ± 0.11 **	6.84 ± 3.11	421.44 ± 221.90 **
	GG	199.61 ± 42.40	0.32 ± 0.27 *	12.86 ± 4.76 *	787.72 ± 109.53
	GC	138.98 ± 33.54	0.50 ± 0.18	5.26 ± 1.93	797.39 ± 173.64
**3**	GSBXD	82.79 ± 15.72	0.81 ± 0.34	12.55 ± 7.46	825.06 ± 96.02
	GSBXD-GS	101.20 ± 17.96	1.33 ± 0.37	7.90 ± 1.17	909.502 ± 476.26
	GSBXD-GC	74.12 ± 25.99	0.31 ± 0.07	16.04 ± 2.62	362.33 ± 40.42
	GSBXD-GG	149.87 ± 24.51 ^#^	0.50 ± 0.28	21.41 ± 8.85	658.98 ± 411.01
**4**	GSBXD	255.17 ± 98.01	0.33 ± 0.18	8.06 ± 1.44	1114.96 ± 346.19
	GSBXD-GS	141.63 ± 39.84	0.28 ± 0.14	9.07 ± 0.87	513.95 ± 271.24
	GSBXD-GC	88.46 ± 18.26	0.81 ± 0.62	7.74 ± 0.74	618.64 ± 129.51
	GSBXD-GG	112.65 ± 25.78 ^#^	0.56 ± 0.27 ^##^	8.73 ± 0.98	785.47 ± 158.14

Data are expressed as mean ± S.D. (*n* = 6). * Difference from corresponding GC group, *p* < 0.05; ** Difference from corresponding GC group, *p* < 0.01; ^#^ Difference from corresponding GSBXD group, *p* < 0.05; ^##^ Difference from corresponding GSBXD group, *p* < 0.01.

#### 2.3.1. Comparison of Pharmacokinetic Profile of Group GC and GG

The *T*_1/2_ and *AUC*_0~t_ values of glycyrrhizinic acid in group GC were significantly decreased compared with those of the group GG as shown in Table 5. We can infer that the herb Gansui can have a have remarkable influence on the pharmacokinetic profile of glycyrrhizinic acid, retard its elimination and enhancing its absorption and bioavailability. After the co-administration of Gancao and Gansui, the *C*_max_ and *T*_1/2_ of liquiritin were larger compared with the administration of Gancao, which showed that Gansui could increase the absorption and prolong the elimination of liquiritin.

#### 2.3.2. Comparison of Pharmacokinetic Profile of Group GC and GSBXD-GS

After GSBXD-GS was administrated to the normal rats, the result of *T*_max_, *C*_max_ and *AUC*_0~t_ of glycyrrhizinic acid were significantly increased, among which *AUC*_0~t_ increased most remarkably (*p* < 0.01). Although the *T*_1/2_ of glycyrrhizinic acid showed no obvious difference in comparison with that in the GC group, its half-life was prolonged. It could be inferred that the herbs Baishao and Banxia may lead to better absorption and higher bioavailability and postpone the peak concentration of glycyrrhizinic acid. 

The herbs Baishao and Banxia also had great effects on liquiritin. We can see from Table 6 that *T*_max_ of liquiritin in group GSBXD-GS was significantly decreased (*p* < 0.01) compared with that in group GC, while the *AUC*_0~t_ of liquiritin increased remarkably. Therefore, it can be speculated that the herbs Baishao and Banxia may lead to higher bioavailability and also advance the arrival of the peak concentration of liquiritin.

#### 2.3.3. Comparison of Pharmacokinetic Profile of Group GSBXD and GSBXD-GG

As shown in Figure 3 and Table 6, the pharmacokinetic parameters of paeoniflorin and albiflorin in the GSBXD group show significant differences in *C*_max_ in comparison with the GSBXD-GG group. The *C*_max_ value of paeoniflorin was significantly decreased (*p* < 0.05). while the *C*_max_ of albiflorin showed a tendency to increase. An obvious decrease of *T*_max_ of albiflorin can also be detected. It is suggested that the herb Baishao administered simultaneously with Gansui and Gancao anti-drug combination could lead to better and quicker absorption of albiflorin, but worse absorption of paeoniflorin.

### 2.4. Influence of Gansui and Gancao Anti-drug Combination and Other Herbs in GSBXD on Pharmacokinetic Profile of Glycyrrhizinic Acid, Liquiritin, Paeoniflorin and Albiflorin

The drug combinations in traditional Chinese recipes could significantly influence the blood concentration and the pharmacokinetic parameters of the individual components after oral administration. In this study, the pharmacokinetics parameter data obtained for glycyrrhizinic acid, liquiritin, paeoniflorin and albiflorin in different groups showed significant differences.

The result indicated that the herb Gansui improved the absorption and slowed down the elimination of the two components in Gancao. It can also lead to better bioavailability of glycyrrhizinic acid. Some published papers [16] have reported that some compounds in the herb Gansui may inhibit P-glycoprotein functions in the intestinal membrane. It is also reported [17] that Gansui can inhibit the activity of the enzyme CYP1A2. Those could be the probable reasons why the main ingredients of Gancao accumulated in the body after Gansui and Gancao were administered simultaneously. The herbs Baishao and Banxia could also lead to the higher bioavailability of glycyrrhizinic acid and liquiritin. The effects of Gansui and Gancao anti-drug combination on the pharmacokinetic profile of Baishao are mainly on *T*_max_ and *C*_max_. We can infer that Gansui and the compatibility of ingredients could lead to better absorption of glycyrrhizinic acid and liquiritin in Gancao, while Gansui and Gancao anti-drug combination could lead to better and quicker absorption of albiflorin, but worse absorption of paeoniflorin. However, all these hypotheses above need to be further investigated.

The study actually has some recognized deficiencies. Firstly, more active components should be chosen in the pharmacokinetic study of GSBXD. Secondly, the active ingredients in the herb Gansui can hardly be detected by using LC-MS/MS.

## 3. Experimental Section

### 3.1. Materials and Reagents

GSBXD includes the following crude drugs: (1) *Euphorbia kansui T.N. Liou ex T.P. Wang.* (2) *Glycyrrhiza uralensis Fisch*, (3) *Paeonia lactiflora Pall*, (4) *Pinellia ternata Breit*. All the materials were purchased from the Fengyuan Phamaceutical Company of Anhui Province (Hefei, China) and authenticated by Jinao Duan. They met the qualitative and quantitative stipulations of the 2010 Chinese Pharmacopoeia. Voucher specimens were deposited in the Herbarium of Nanjing University of Chinese Medicine, Nanjing, China. Acetonitrile and formic acid were HPLC-grade from Merck (Darmstadt, Germany) and deionized water was purified by an EPED super purification system (Eped, Nanjing, China). The reference compounds liquiritin (111610-201106), paeoniflorin (110736-201337), albiflorin (110736-200833) were purchased from the Chinese National Institute of Pharmaceutical and Biological Products (Beijing, China). glycyrrhizinic acid (220863) were purchased from Nanjing Spring-Autumn Biological Engineering Co., Ltd (Nanjing, China). All other reagents were obtained from Sinopharm Chemical Reagent Co., Ltd. (Nanjing, China), unless otherwise stated.

### 3.2. Animals

All experiments were performed with male Wistar rats, weighing 220–250 g, obtained from the Vital River Experimental Animal Co., Ltd., Beijing, China. They were kept in plastic cages at 22 ± 2 °C with free access to pellet food and water. Animal welfare and experimental procedures were carried out in accordance with the guide for the care and use of laboratory animals (National Research Council, Washington, DC, USA). Committee for the Update of the Guide for the Care and Use of Laboratory Animals (2011) and related ethical regulations of Nanjing University of Chinese Medicine.

### 3.3. Chromatographic Conditions

Chromatographic analysis was performed on a Waters Acquity UPLC system (Waters Corp., Milford, MA, USA), consisting of a binary pump solvent management system, an online degasser, and an auto-sampler. An ACQUITY UPLC BEH C 18 (100 mm × 2.1 mm, 1.7 μm) column was applied for all analyses. And the column temperature was maintained at 35 °C. The mobile phase was composed of (A) formic acid aqueous solution (0.1%) and (B) acetonitrile using a gradient elution of 10%–50% B at 0–3 min, 50%–95% B at 3–4 min, 95% B at 4–5 min, 95%–100% B at 5–5.2 min.

### 3.4. Mass Conditions

Mass spectrometry detection was performed using a Xevo Triple Quadrupole MS (Waters Corp.) equipped with an electro spray ionization source (ESI). The ESI source was set in both positive and negative ionization mode. The parameters in the source were set as follows: capillary voltage 3.0 kV; source temperature 150 °C; desolvation temperature 550 °C; cone gas flow 50 L/h; desolvation gas flow 1000 L/h. The analyte detection was performed by using multiple reaction monitoring (MRM). The cone voltage and collision energy were optimized for each analyte and selected values are given in Table 6. All data collected in centroid mode were acquired using Masslynx 4.1 software (Waters Corp.) and post-acquisition quantitative analysis was performed using the TargetLynx program (Waters Corp.). 

**Table 6:** Precursor/production pairs and parameters for MRM of compounds used in this study.

Analyte	Retention Time(min)	[M+H]^+^ (*m/z*)	MRM Transitions (Precursor-product)	Cone Voltage(V)	Collision Energy (eV)
Glycyrrhizinic acid	3.48	823.35	823.35→453.35	18	30
Liquiritin	1.89	416.99	416.99→255.14	28	20
Paeoniflorin	1.77	502.99	502.99→89.11	40	22
Albiflorin	1.63	480.99	480.99→105.10	16	30

### 3.5. Preparation of GSBXD and Omitted Ingredients in GSBXD

Raw materials of *Glycyrrhiza uralensis Fisch* (Gancao), *Paeonia lactiflora Pall* (Baishao), *Pinellia ternata Breit* (Banxia) in a weight ratio of 10:15:9 (50, 75 and 45 g) were crushed into small pieces. The mixture was refluxed with 1.7 L water for 1 h and with 1.36 L water for 1 h. The filtrates were combined and concentrated below 70 °C to obtain a certain volume at the ratio of 1.5:1 (w/w, weight of all constituting herbs and the extract filtrates) under vacuum. Powder of *Euphorbia kansui T. N. Liou ex T. P. Wang.* (Gansui) (1.5 g) was added into the extract and then blended well. The same method was used to prepare GSBXD minus Gancao (GSBXD-GC); GSBXD minus Gansui (GSBXD-GS); GSBXD minus Gancao and Gansui (GSBXD-GG); the extract of Gancao and Gansui (GG) and the extract of Gancao (GC). The extracts contained 19.21, 18.73, 46.53, 15.82 μg/mL of compounds **1–4** in GSBXD; 0, 0, 51.03, 17.88 μg/mL of compounds **1–4** in GSBXD-GC; 19.21, 18.73, 46.53, 15.82 μg/mL of compounds **1–4** in GSBXD-GS; 0, 0, 51.03, 17.88 μg/mL of compounds **1–4** in GSBXD-GG; 20.03, 17.09, 0, 0 μg/mL of compounds **1–4** in GG, 20.03, 17.09, 0, 0 μg/mL of compounds **1–4** in GC.

### 3.6. Preparation of Calibration Standards and Quality Control Samples

A standard stock solution mixture containing four compounds was prepared in methanol with a final concentration of 24.5 μg/mL for glycyrrhizic acid (**1**), 22.1 μg/mL for liquiritin (**2**), 22.8 μg/mL for paeoniflorin (**3**), and 28.3 μg/mL for albiflorin (**4**), respectively. The mixture stock solution was serially diluted with methanol to provide working standard solutions of the desired concentrations. The IS stock solutions (24.6 μg/mL for diphenhydramine—IS for positive ionization mode and 23.8 μg/mL for chloromycetin—IS for negative ionization mode) were also prepared in methanol. IS working solutions (1215 ng/mL for diphenhydramine, 476 ng/mL for chloromycetin) were prepared by diluting the stock solution with methanol. Calibration samples were prepared by mixing solutions of standard mixture, IS and methanol with rat blank plasma to obtain final concentrations in the range of 19.14–2450 ng/mL for glycyrrhizic acid, 4.32–2210 ng/mL for liquiritin, 17.81–2280 ng/mL for paeoniflorin, 11.05–2830 ng/ml for albiflorin, and 1215 ng/mL for diphenhydramine (IS for positive ionization mode) and 476 ng/mL for chloromycetin (IS for negative ionization mode) for IS respectively. All solutions were stored at −20 °C before use. Quality control (QC) samples were also prepared in the same way (19.14, 306.25, 1225 ng/mL for glycyrrhizic acid, 4.32, 138.13, 1105 ng/mL for liquiritin, 17.81, 142.5, 1140 ng/mL for paeoniflorin, 11.05, 176.88, 1415 ng/mL for albiflorin) at low, middle and high concentrations.

### 3.7. Validation Procedures

The specificity of the method was evaluated by preparing and analyzing six different batches of rat plasma to investigate the potential interferences at the LC peak region for analytes and IS. The rat plasma chromatograms were compared with those obtained with a sample at the concentration of lower limit of quantification (LLOQ).The signal intensity at this concentration was at least five times higher than that of blank plasma.

The linearity of each calibration curve was determined by plotting the peak area ratio (y) of analytes to IS versus the nominal concentration (x) of analytes with weighted (1/x^2^) least square linear regression.

Accuracy and intra- and inter-day precision were estimated by analyzing three QC samples (five samples for each) at low, middle and high concentrations on the same day and on three consecutive validation days respectively. The precision was evaluated by relative standard deviation (RSD %) and accuracy by (mean measured concentration/spiked concentration) × 100%.

Extraction recovery was assessed by comparing the peak responses of three QC samples (five samples for each) with the responses of analytes from standard solutions spiked in post-extracted black plasma at equivalent concentrations. 

Matrix effect was measured via comparison of the peak responses obtained from samples where the extracted matrix was spiked with standard solutions to those obtained from neat standard solutions at equivalent concentrations.

Three QC samples (five samples for each) were tested for pre-treatment, post-treatment, three freeze-thaw cycles and long-term stabilities. Pre-treatment stability was assessed by exposing QC samples at room temperature for 4 h. Post-treatment stability was evaluated by placing QC samples in the auto-sampler at 4 °C for 24 h. For freeze–thaw cycle stability assessment, QC samples were repeatedly frozen and thawed for three cycles from −80 °C to 20 °C. Long-term stability was carried out via placing QC samples at −80 °C for 2 weeks.

### 3.8. Pharmacokinetic Studies

For pharmacokinetic studies, Wistar rats were divided into ten groups (*n* = 6 per group). Rats in the normal and model GSBXD groups were administered GSBXD at a dose of 15 mL/kg intragastrically. In the normal and model GSBXD-GC groups, GSBXD-GC (12 mL/kg) was administrated. In the normal and model GSBXD-GS groups, GSBXD-GS (15 mL/kg) was administrated. In the normal and model GSBXD-GC-GS groups, GSBXD-GC-GS (12 mL/kg) was administrated. In the normal and model GG groups, GG (7.5 mL/kg) was administrated. Blood samples were collected at specific time points before (0 min) and after oral administration (5, 10, 20, 40, 60, 120, 240, 360, 480, 600 and 1440 min). A total of 720 blood samples were collected. All the blood samples were immediately centrifuged at 2500 rpm for 10 min to obtain plasma, which was labeled and frozen at −80 °C until analysis. Blank plasma was obtained from the rats without oral administration and was used to investigate the assay development and validation.

### 3.9. Statistical Analysis

To determine the pharmacokinetic parameters of compounds **1–4** in different groups, concentration–time data were analyzed by DAS 3.2 software (Mathematical Pharmacology Professional Committee of China, Shanghai, China, 2011). Data were expressed as the mean 7 standard deviation (S.D.) with triplicate measurements. The identification of significances between different groups was carried out with Student’s t-test. A P value < 0.05 was considered statistically significant.

## 4. Conclusions

In this paper, a rapid, selective and specific LC-MS/MS method for the simultaneous analysis of four components in rat plasma using a 6.0 min simple chromatographic run was developed for the first time. The results obtained from this study demonstrated that Gansui and the compatibility of ingredients could lead to better absorption of glycyrrhizinic acid and liquiritin in Gancao, while Gansui and Gancao anti-drug combination could lead to better and quicker absorption of albiflorin, but worse absorption of paeoniflorin. The obtained knowledge might contribute to the rationality of the clinic use of GSBXD and also reveal the compatibility conditions of the Gansui and Gancao anti-drug combination.
